# Cerebral Hemodynamic Responses During Dynamic Posturography: Analysis with a Multichannel Near-Infrared Spectroscopy System

**DOI:** 10.3389/fnhum.2015.00620

**Published:** 2015-11-17

**Authors:** Hiromasa Takakura, Hisao Nishijo, Akihiro Ishikawa, Hideo Shojaku

**Affiliations:** ^1^Department of Otorhinolaryngology, Head and Neck Surgery, Graduate School of Medicine and Pharmaceutical Sciences, University of Toyama, Toyama, Japan; ^2^System Emotional Science, Graduate School of Medicine and Pharmaceutical Sciences, University of Toyama, Toyama, Japan; ^3^R&D Department, Medical Systems Division, Shimadzu, Co., Ltd., Kyoto, Japan

**Keywords:** NIRS, dynamic posturography, sensory conflict, vestibular cortices, supplementary motor cortex, spatial reference frames, premotor cortex, posterior parietal cortex

## Abstract

To investigate cortical roles in standing balance, cortical hemodynamic activity was recorded from the right hemisphere using near-infrared spectroscopy (NIRS) while subjects underwent the sensory organization test (SOT) protocol that systematically disrupts sensory integration processes (i.e., somatosensory or visual inputs or both). Eleven healthy men underwent the SOT during NIRS recording. Group statistical analyses were performed based on changes in oxygenated hemoglobin concentration in 10 different cortical regions of interest and on a general linear analysis with NIRS statistical parametric mapping. The statistical analyses indicated significant activation in the right frontal operculum (f-Op), right parietal operculum (p-Op), and right superior temporal gyrus (STG), right posterior parietal cortex (PPC), right dorsal and ventral premotor cortex (PMC), and the supplementary motor area (SMA) under various conditions. The activation patterns in response to specific combinations of SOT conditions suggested that (1) f-Op, p-Op, and STG are essential for sensory integration when standing balance is perturbed; (2) the SMA is involved in the execution of volitional action and establishment of new motor programs to maintain postural balance; and (3) the PPC and PMC are involved in the updating and computation of spatial reference frames during instances of sensory conflict between vestibular and visual information.

## Introduction

For the maintenance of upright position stability, the convergence of sensory information from multiple inputs, such as vestibular, somatosensory, and visual information that provides signals for the detection of the body’s position in space, is important. The neural systems that regulate postural orientation and equilibrium continually integrate a large array of sensory inputs and coordinate multiple motor outputs to muscles throughout the body (Lockhart and Ting, 2007; Ting, 2007). The neural mismatch (i.e., sensory conflict) among these three sensory inputs elicits vertiginous sensation and postural instability (Brandt, 1999). For example, sudden unilateral loss of vestibular function, such as with vestibular neuritis, causes immediate ataxia and severe postural instability, and some patients suffer from residual instability after the process of vestibular compensation (Horak, 2009; Peterka et al., 2011). Motion sickness is generated either by unfamiliar body ­accelerations or by intersensory mismatch between vestibular and visual stimuli and induces an unpleasant illusion of movement (Brandt, 1999; Keshavarz et al., 2015). In nature, sensory signals of different modalities are in general redundant and plastic to ensure delivery of appropriate environmental information to the central nervous system (CNS) (Day and Guerraz, 2007). In a situation of sensory conflict, one distorted or unavailable sensory modality is chosen over the other modalities to maintain postural balance (Dickstein et al., 2001) or can even override all other modalities (Diedrichsen et al., 2007). Thus, it is now generally acceptable that visual, vestibular, and somatosensory inputs are dynamically reweighted to maintain upright stance as environmental or nervous system conditions change, and this phenomenon is referred to as “sensory reweighting” (Assländer and Peterka, 2014; Logan et al., 2014). Previous functional imaging studies reported that activation or inhibition in vestibular cortices was found according to cognitive demands during self-motion perception and suggested that cortical cognitive processes are involved in sensory reweighting in a situation of sensory conflict (Brandt et al., 1998; Deutschländer et al., 2002; Bense et al., 2005). These results suggest that vestibular cortices might be one of the key regions for cortical processing to maintain postural balance in sensory conflict. However, positron emission tomography (PET) or functional magnetic resonance imaging (fMRI) was used in those studies, and participants were not required to maintain a standing balance. Therefore, it is unclear how vestibular cortices are involved in maintaining postural balance in a situation of sensory conflict.

The traditional view suggests that balance control occurs at a very automatic level, primarily involving the spinal cord and brainstem. However, there is growing evidence that the cerebral cortex and cognitive processing are involved in controlling specific aspects of balance (Maki and McIlroy, 2007). For example, a high incidence of falls has been found in patients with cortical lesions or cognitive deficits (De Vincenzo and Watkins, 1987; Mion et al., 1989; Vlahov et al., 1990; Rapport et al., 1993). Furthermore, a response to an unpredictable perturbation requires an online response modification based on a subject’s intentions (i.e., online use of cortical influence) (Jacobs and Horak, 2007). Recent human studies suggested the involvement of cortical structures, such as the supplementary motor area (SMA), anterior cingulate cortex (ACC), and posterior parietal cortex (PPC), during control of balance and posture (Mihara et al., 2008; Varghese et al., 2014; Hülsdünker et al., 2015). Previous studies have suggested that the SMA is involved in establishing motor programs (Picard and Strick, 1996), preparation for foot movement (Sahyoun et al., 2004), human locomotion (Miyai et al., 2001), and human balance control (Mihara et al., 2008). The ACC is involved in error detection and processing during balance control (Sipp et al., 2013; Hülsdünker et al., 2015), and the PPC receives multimodal inputs from somatosensory, vestibular, and visual systems. The PPC is a key region for sensory information processing and sensorimotor transformation processes (Reichenbach et al., 2014). Thus, involvement of the frontal and parietal cortices might be crucial during balance control. However, it is unclear how the frontal, especially the motor-related, and posterior parietal cortices are involved in postural balance activities in sensory conflict.

Computerized dynamic posturography (CDP), which was originally developed by Nashner (1970), Nashner et al. (1989) provides an objective assessment of balance control and postural stability under dynamic test conditions. As a part of CDP, the sensory organization test (SOT) protocol systematically disrupts sensory integration processes (i.e., somatosensory or visual inputs or both) while measuring a subject’s ability to maintain equilibrium. The SOT has been used extensively in both research and clinical practice (Black et al., 1995; Di Fabio, 1995; Cohen et al., 1996; Shahal et al., 1999; Allum et al., 2002; Ferber-Viart et al., 2007; Wrisley et al., 2007; Ray et al., 2008; Honaker et al., 2009; Leitner et al., 2009). In the SOT, information received from the patient’s eyes, feet, and joints that is useful for the maintenance of equilibrium can be effectively canceled by means of the calibrated “sway referencing” of the support surface (floor) on which the patient stands and/or the visual surround around the patient (i.e., the support surface and/or the visual surround tilts to directly follow the patient’s anterior–posterior body sway so that sensory organs do not detect the changes). By controlling the sensory (visual and proprioceptive) information through sway referencing with the eyes open or closed, the SOT protocol systematically eliminates the efficacy of visual and/or proprioceptive information. Thus, the SOT protocol can create different scenarios of sensory conflict.

Near-infrared spectroscopy (NIRS), one of the functional neuroimaging techniques, detects differences in the absorption spectra of oxygenated hemoglobin (Oxy-Hb) versus deoxygenated hemoglobin (Deoxy-Hb) in the near-infrared spectrum range. Among the various neuroimaging techniques, functional NIRS (fNIRS) can non-invasively facilitate the measurement of task-related cortical responses (Jöbsis, 1977; Colacino et al., 1981). In terms of the subject’s motion, the imaging technique is relatively robust. Accordingly, fNIRS is suitable for investigating the cortical control of postural balance (Mihara et al., 2008).

In the present study, we recorded brain hemodynamic activity during the control of postural balance in the SOT using a multichannel NIRS system to investigate the cortical cognitive processes during instances of sensory conflict in postural balance activities. A previous study investigated hemodynamic changes during CDP using fNIRS and reported that there were bilateral activations in the temporal-parietal areas [superior temporal gyrus (STG) and supramarginal gyrus (SMG)] when both vision and proprioceptive information were degraded (Karim et al., 2013). However, the recorded cortical regions were limited to the bilateral temporal areas, and the involvement of the motor and parietal cortices in the control of postural balance during CDP was unknown. In our study, we decided to record hemodynamic responses from more extended cortical areas including the temporal, frontal, and parietal cortices in the right hemisphere.

Analyses of spontaneous hemodynamic fluctuations observed in fMRI or NIRS have revealed temporal correlations in signal changes between widely separated brain regions during the resting state, termed “resting-state functional connectivity” (Liu et al., 2008; Lu et al., 2010; Sasai et al., 2011, 2012). Functional connectivity is characterized in fMRI by a temporal correlation between two raw time series with “low frequency (0.01–0.1 Hz), which is separable from respiratory (0.1–0.5 Hz) and cardiovascular (0.6–1.2 Hz) signal frequencies (Cordes et al., 2001) and reflects a level of functional communication between regions (van den Heuvel and Hulshoff Pol, 2010). A recent study showed that NIRS can collect information regarding resting-state networks defined in fMRI (Sasai et al., 2012). Furthermore, other recent studies investigated task-relevant changes of functional connectivity during working memory tasks (Sala-Llonch et al., 2012) and during motor tasks (Bajaj et al., 2014). We also analyzed changes of functional connectivity between the frontal, parietal, and temporal cortices during postural balance in a situation of sensory conflict.

We would expect the following changes in cortical activities during instances of sensory conflict in postural balance: (1) when only vestibular inputs are normal, but somatosensory and visual inputs are disrupted or absent in SOT conditions 2–6, sensory reweighting to the vestibular input would be induced, and activations in the vestibular cortices would be stronger along with increases in conflict due to disrupted or absent inputs (i.e., SOT5 and 6); (2) the frontal and parietal cortices would activate more strongly along with increases in conflict due to disrupted or absent inputs (i.e., SOT5 and 6); and (3) in functional connectivity analyses, network correlations among the vestibular, frontal, and parietal cortices would be increased during postural balancing in sensory conflict. The magnitudes and patterns of network activities in each SOT would be different.

## Materials and Methods

### Subjects and Tasks

Eleven healthy men [aged 33.4 ± 7.4 (mean ± STD) years, all right handed] were enrolled in this study. All subjects were treated in strict compliance with the Declaration of Helsinki and the U.S. Code of Federal Regulations for the protection of human subjects. The experiments were conducted with the full consent of each subject using a protocol approved by the ethical committee for human experiments of the University of Toyama.

### Tasks

Each subject performed the EquiTest^®^ SOT (Version 5.08b, NeuroCom International, Inc., Clackamas, OR, USA), which provides an extremely sensitive objective assessment of the main sensory systems involved in balance and stability. The SOT protocol sets up six conditions that systematically disrupt the sensory integration processes (i.e., proprioceptive or visual inputs or both) while measuring a subject’s ability to maintain equilibrium. The six sensory conditions evaluate the relative contributions of vision and vestibular and somatosensory inputs in the balance function (Table 1). Under SOT condition 1 (SOT1), all three sensory systems were operational while the participant stood on a fixed platform with his eyes open, and a baseline measure of stability was obtained. SOT2 was the same as the SOT1, except that the eyes were closed. SOT3 was similar to SOT1, but the visual surround moved to track the participant’s sway, which provided inaccurate orientation cues. Under SOT4, the subject stood with his eyes open and the visual surround fixed, but the platform moved in response to his/her sway so that the ankle joints did not bend along with the sway, providing an inaccurate proprioceptive input to the brain. SOT5 was identical to SOT4, except that the eyes were closed, so that only the vestibular system was fully operational. SOT6 was the same as SOT4, except that the visual surround moved along with the participant’s sway, and thus both vision and proprioception were compromised, leaving the vestibular system as the only reliable sensory source.

**Table 1:** **The six experimental conditions of the Sensory Organization Test (SOT)**.

SOT	Vision	Platform	Visual surround	Accurate sensory inputs	Compromised sensory inputs
1	Eyes open	Fixed	Fixed	Vest, Vis, Som	None
2	Eyes closed	Fixed	Fixed	Vest, Som	None
3	Eyes open	Fixed	Sway-referenced	Vest, Som	Vis
4	Eyes open	Sway-referenced	Fixed	Vest, Vis	Som
5	Eyes closed	Sway-referenced	Fixed	Vest	Som
6	Eyes open	Sway-referenced	Sway-referenced	Vest	Vis, Som

*Vest, vestibular; Vis, visual; Som, somatosensory*.

In this study, each subject completed five trials under each condition. Each trial lasted 20 s, and each intertrial interval was set for more than 60 s. During the intertrial interval, each subject stood on a fixed platform with his eyes open and with the three sensory systems operational (i.e., under the same conditions as SOT1). After completing three trials under the six SOT conditions, the subjects rested for a few minutes to recover from their fatigue. Thus, this task involved 30 trials overall and typically lasted about 60 min.

### fNIRS Recording

**Figure 1:**
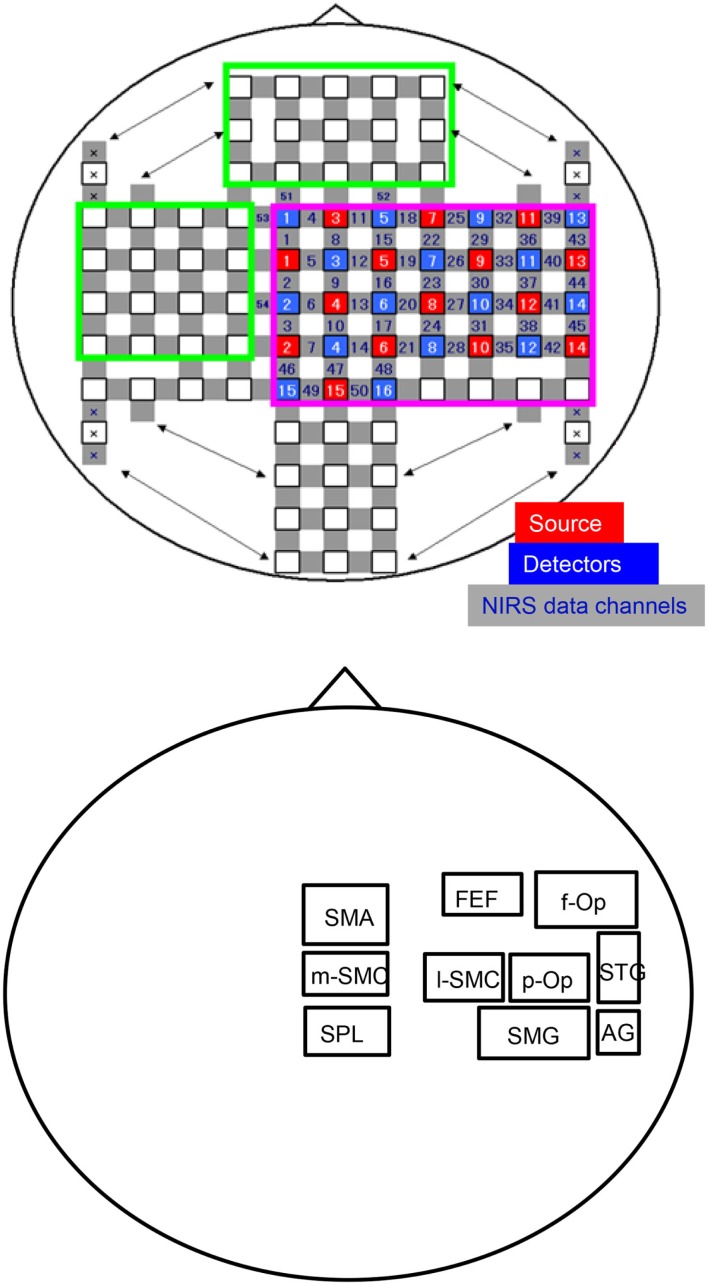
**An illustration of the arrangement of the optodes (sources and detectors) and recording channels (A) and a schema of the 10 regions of interest (ROIs) for the group-averaged NIRS data analysis (B)**. f-Op, right frontal operculum/inferior frontal gyrus; p-Op, right parietal operculum; FEF, frontal eye field; SMG, right supramarginal gyrus; AG, right angular gyrus; STG, right superior temporal gyrus; l-SMC, lateral part of the sensorimotor cortex in the right hemisphere; m-SMC, medial part of the sensorimotor cortex; SPL, superior parietal lobule; SAC, somatosensory association cortex; SMA, supplementary motor area.

A head cap (FLASH-PLUS; Shimadzu Co., Ltd., Japan) for fNIRS was placed on the subject’s head. The two optodes (the source and the detector) of the fNIRS-imaging system (OMM-3000; Shimadzu Co., Ltd.; 15 sources, 16 detectors, and 50 channels) were then attached to the parietal and right temporal parts of the head cap (Figure 1A). We could not record hemodynamic responses from whole cortical areas with our NIRS system. Therefore, we decided to record them from the right hemisphere in this study because a previous study indicated the dominance for vestibular cortical functions in the non-dominant hemisphere, i.e., in the right hemisphere of right-handed subjects (Dieterich et al., 2003).

The distance between the NIRS source and the detector was set at 3 cm, and the optodes were positioned crosswise from each other. Hemodynamic responses were measured at the midpoints between the source and detector, which were called “NIRS channels.” Three different wavelengths (780, 805, and 830 nm), each with a pulse width of 5 ms, were used to detect hemodynamic responses. The details of the head cap and the systems have been described previously (Takeuchi et al., 2009; Takamoto et al., 2010; Takakura et al., 2011). After the recording, the three-dimensional (3-D) locations of the optodes were measured by a 3-D Digitizer (Nirtrack; Shimadzu Co., Ltd.) in reference to the nasion and bilateral external auditory meatus.

To estimate the anatomical locations of the optodes and the NIRS channels, we used the “Spatial registration of NIRS channel locations” function of the NIRS-SPM (statistical parametric mapping) Version 3.0 software, which is an SPM5- and MATLAB-based software package for the statistical analysis of NIRS signals freely downloadable from http://bisp.kaist.ac.kr/NIRS-SPM (Ye et al., 2009). Using the “Stand alone” option [without using magnetic resonance imaging (MRI) images], we estimated the spatial representation of the NIRS channel locations on the normalized brain surface (Friston et al., 1995; Ashburner et al., 1997; Ashburner and Friston, 1999) using a Montreal Neurological Institute (MNI) brain template, which corresponds to the space described by Talairach and Tournoux (1998). In each subject, the estimated locations of the NIRS channels were anatomically labeled using the 3-D digital brain atlas known as “Talairach daemon” (Lancaster et al., 2000), which is incorporated into the NIRS-SPM.

The MRI images of the heads of some subjects were used later to reconstruct a realistic 3-D head model, and the location of each NIRS channel and topographical maps of the changes in Oxy-Hb concentration were superimposed on the surface of the 3-D MRI reconstructed brain of those subjects with Fusion 3-D imaging software (Shimadzu Co., Ltd.).

### Regions of Interest

**Figure 2:**
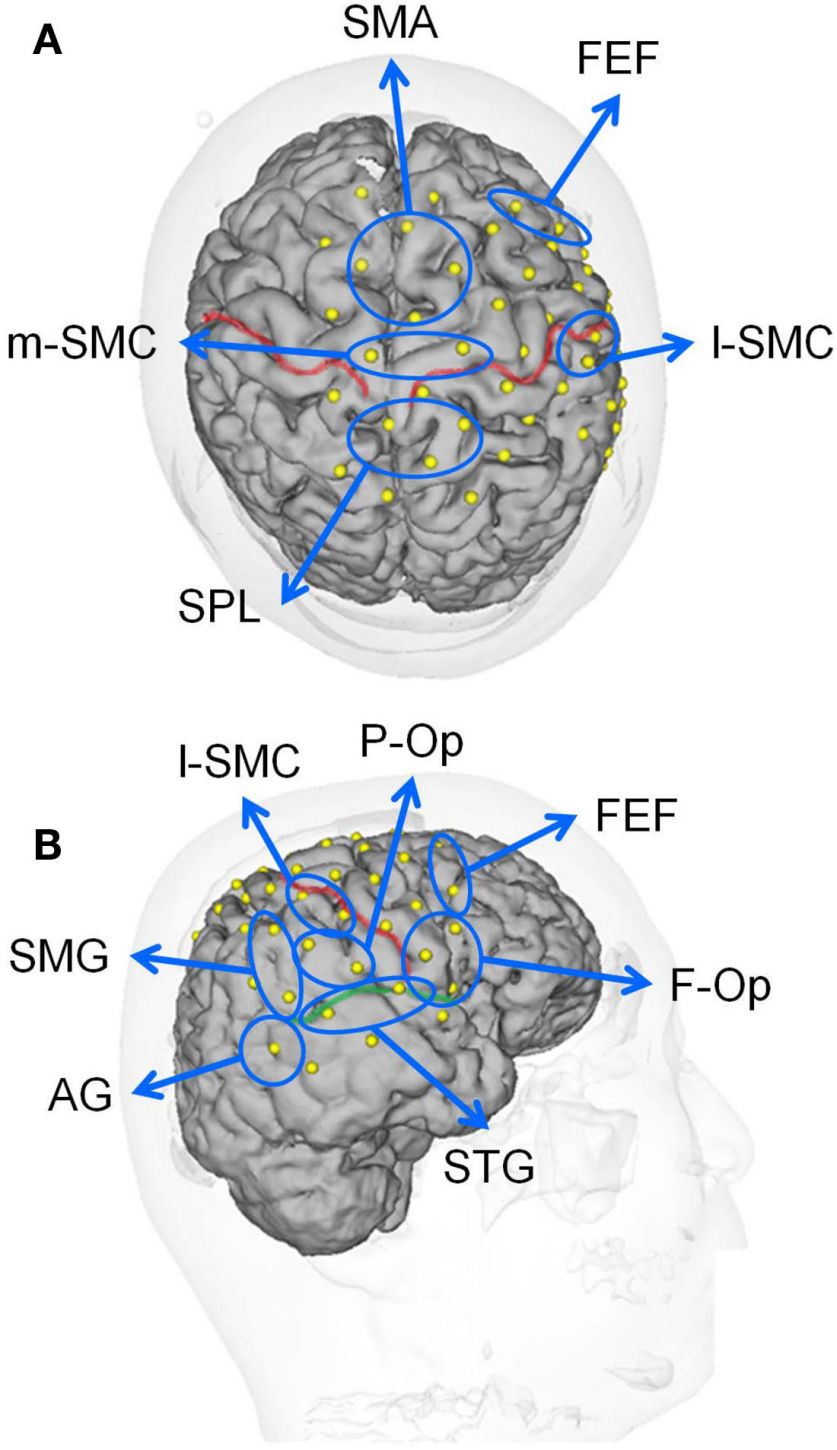
**Locations of the recording channels and 10 regions of interest (ROIs) on a 3-D MRI reconstruction of the brain of one of the subjects**. **(A,B)** indicate the top and right views of the brain, respectively. The red and green lines indicate the central sulcus and right Sylvian fissure, respectively. f-Op, right frontal operculum/inferior frontal gyrus; p-Op, right parietal operculum; FEF, frontal eye field; SMG, right supramarginal gyrus; AG, right angular gyrus; STG, right superior temporal gyrus; l-SMC, lateral part of the sensorimotor cortex in the right hemisphere; m-SMC, medial part of the sensorimotor cortex; SPL, superior parietal lobule; SAC, somatosensory association cortex; SMA, supplementary motor area.

In this study, we divided the NIRS channels into several groups that covered the different cortical regions of interest (ROIs) (Figure 1B). We created 10 ROIs on the brain surface: the right frontal operculum/inferior frontal gyrus (f-Op), the right parietal operculum (p-Op), the frontal eye field (FEF), the right SMG, the right angular gyrus (AG), the right STG, the lateral part of the sensorimotor cortex in the right hemisphere (l-SMC), the medial part of the sensorimotor cortex (m-SMC), the superior parietal lobule (SPL), and the SMA. Each ROI included two to four NIRS channels according to the number of anatomically estimated channel locations in the MNI template. A schematic presentation of these brain regions is shown in Figure 2.

One of our great interests is how the vestibular cortex activates under each SOT condition. It is now generally accepted that there is no primary vestibular cortex that exclusively receives vestibular afferents, and several multimodal sensory areas have been identified as vestibular cortical areas (Brandt and Dieterich, 1999; Lopez and Blanke, 2011). A systematic review of human imaging studies on the vestibular cortex indicated that the posterior insula and temporoparietal junction, anterior insula, PPC, precuneus, middle and superior frontal gyri, somatosensory cortex, cingulate gyrus, and hippocampus were activated by various vestibular stimulations (Lopez and Blanke, 2011). Therefore, we set up the ROIs (i.e., p-Op, STG, AG, f-Op, SMG, SPL, FEF, and l-SMC) to cover these cortical areas except for the deeper parts of the brain (i.e., the cingulate cortex and hippocampus). The m-SMC was selected to compare the activities with that in the l-SMC. We also selected the SMA as a ROI because our second interest is how the frontal motor cortices, especially the SMA, and parietal cortex activate under each SOT condition.

### Data Analysis

#### Analysis of EquiTest^®^ Posturography Data

To evaluate the subject’s postural stability during each SOT condition, the equilibrium score was used in this study. The subject’s sway was calculated from the maximum anterior and posterior centers of gravity displacements over the 20-s trial period. Maximum displacement without losing balance was assumed to be within a range of 12.5° (6.25° anterior, 6.25° posterior). The equilibrium score was calculated according to the following formula: equilibrium score = {12.5 − [(the maximum anterior sway angle in degrees during a trial) − (the maximum posterior sway angle in degrees during the same trial)]} × 100/12.5 (Chaudhry et al., 2004). Equilibrium scores were then expressed as percentages, with 0 indicating sway exceeding the limits of stability and 100 indicating perfect stability. The mean equilibrium score for five trials under each SOT condition was calculated for each subject. The data of mean equilibrium scores were analyzed by one-factorial repeated-measures ANOVA with SOT as the within-subject variable. The level of statistical significance was set at *P* < 0.05. These statistical analyses were performed with a commercial statistical package (IBM SPSS Statistics Version 22.0, IBM Corporation).

#### Analysis of Hemodynamic Responses

In the present study, we focused on changes in Oxy-Hb concentration, which has been reported to be sensitive to neuro-hemodynamic relationships (Hoshi et al., 2001; Strangman et al., 2002; Yamamoto and Kato, 2002). The NIRS data were summed and averaged with reference to the onset of each SOT trial. Furthermore, the effect size was calculated to adjust for the influences of different path length factors among the different subjects and cortical regions (Schroeter et al., 2003; Suzuki et al., 2008). The effect sizes of the hemodynamic responses were calculated according to the following formula: effect size = [(mean Oxy-Hb levels during each SOT for 20 s) − (mean Oxy-Hb levels during the rest period for 20 s before the start of each SOT)]/(standard deviation of Oxy-Hb levels during the rest period of 20 s before the start of each SOT). For each channel, the effect sizes of five trials were averaged. The effect sizes in all of the NIRS channels within the same ROI were averaged in each subject for each SOT condition.

#### Statistical Analysis of Changes in Oxy-Hb

The data of the effect sizes in Oxy-Hb concentration were analyzed by two-factorial ANOVA with repeated-measures [the SOT condition (SOT1-6: within-subject factors) × the ROI (within-subject factor)]. The Greenhouse–Geisser adjustment to the degrees of freedom was applied to all ANOVA to correct for the violation of the assumption of sphericity. When significant interactions were found, *post hoc* tests were performed using tests for the simple effect of one-factorial ANOVA and/or the Fisher protected least significant difference test. The level of statistical significance was set at *P* < 0.05. These statistical analyses were also performed with IBM SPSS Statistics Version 22.0 (IBM Corporation).

#### Statistical Analysis Based on the General Linear Model

Group statistical analyses using NIRS-SPM were also performed for each SOT condition. Based on the general linear model (GLM) and Sun’s tube formula/Lipschitz–Killing curvature-based expected Euler characteristics, NIRS-SPM not only provides activation maps of Oxy-Hb, Deoxy-Hb, and total hemoglobin but also allows for super-resolution activation localization. More details are described in Ye et al. (2009) and Li et al. (2012). The GLM is a statistical linear model that explains data as a linear combination of an explanatory variable plus an error term. Because the GLM measures the temporal-variational pattern of signals rather than their absolute magnitude, it is more robust in many cases, even for those signals with an incorrect diffusion path length factor or with severe optical signal attenuation due to scattering or poor contact (NIRS-SPM users’ guide). The level of statistical significance was set at *P* < 0.05 (uncorrected) in this study.

To estimate the 3-D localization of the activated cortical regions on the normalized brain from the results of the group analysis, the following analyses were also performed: (i) a total of 550 anatomical locations of NIRS channels were estimated for all subjects using the “Spatial registration of NIRS channel locations” function of NIRS-SPM; (ii) the *T*-values at the coordination of the NIRS channels under all SOT conditions were estimated for all subjects from the *T*-value map of group statistical analyses using NIRS-SPM; (iii) all of the estimated locations of the NIRS channels were anatomically labeled using the 3-D digital brain atlas known as the Talairach daemon (Lancaster et al., 2000), which is incorporated into NIRS-SPM; and (iv) the NIRS channel that showed the highest *T*-value in the same anatomical brain region was postulated to be the representative 3-D location of the region.

#### Analysis of Functional Connectivity Between Activated Cortical Regions in SOTs

Functional connectivity was analyzed to elucidate the cortical network activity in the SOTs. First, all channels in activated cortical regions indicated in the group statistical analysis using NIRS-SPM were selected using the Talairach daemon. A band-pass Fourier filter (0.01–0.1 Hz) in the raw time series of the Oxy-Hb signals was used to remove long-term baseline drift and higher frequency cardiac or respiratory activity (Cordes et al., 2001; Lu et al., 2010). After these processes, for a given pair of ROIs in each subject, we calculated cross-correlations of NIRS signals between all possible channel pairs because each ROI included multiple channels. Then, the highest value was used as a coefficient between the given pair of ROIs. We calculated mean coefficients among all subjects in each pair of brain regions and mapped mean coefficients >0.6 (Sasai et al., 2011) as significant functional connectivity in each SOT.

For statistical comparison, all coefficients were converted to *z* scores using Fischer’s *z* transformation. The *z* score data were analyzed by two-factorial ANOVA with repeated-measures [the SOT condition (SOT1-6: within-subject factors) × the pair of regions (within-subject factors)]. When significant interactions were found, *post hoc* tests were performed using tests for the simple effect of one-factorial ANOVA and/or the Fisher protected least significant difference test. The level of statistical significance was set at *P* < 0.05. These statistical analyses were also performed with IBM SPSS Statistics Version 22.0 (IBM Corporation).

## Results

### Posturography Data in SOT

**Figure 3:**
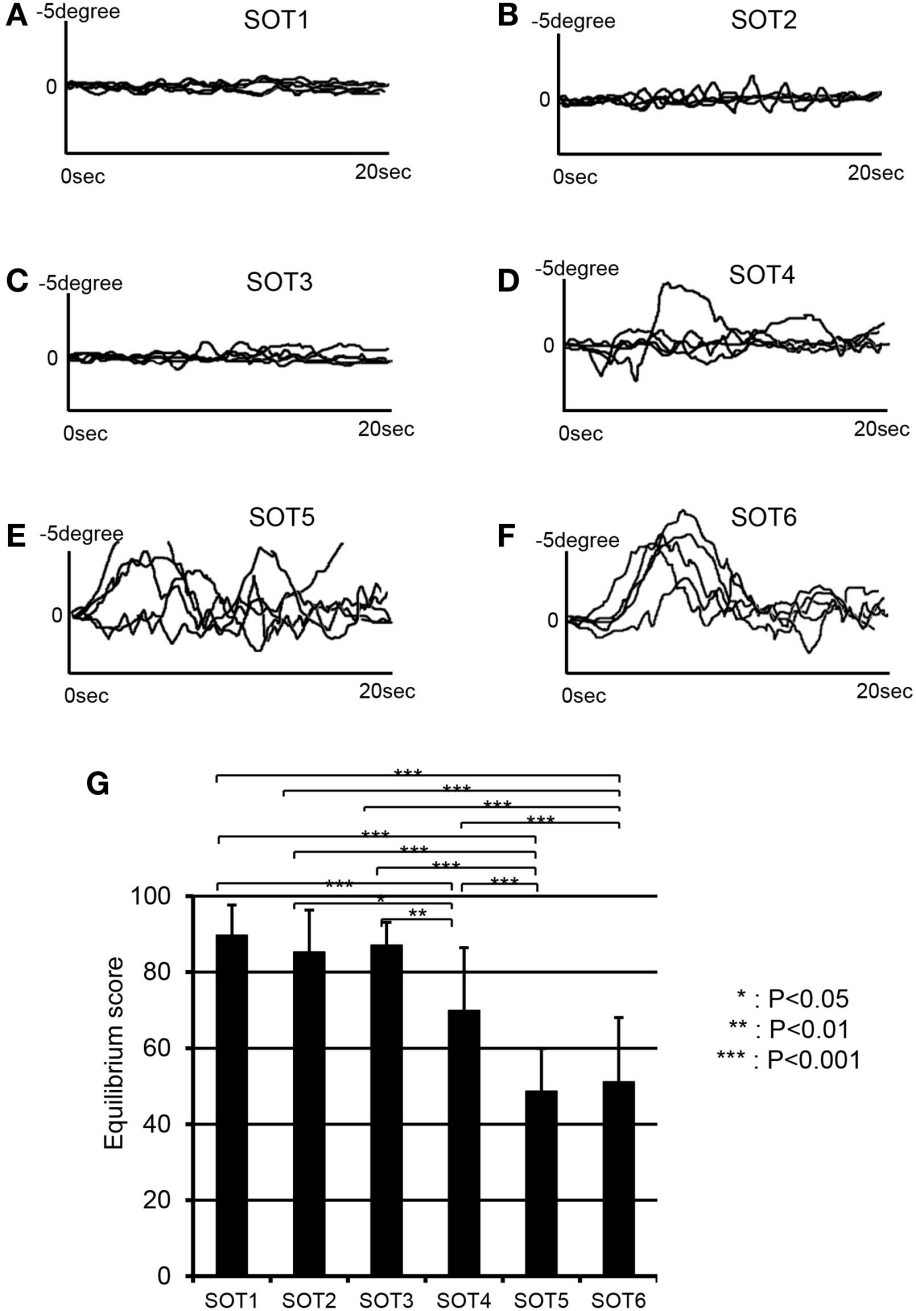
**EquiTest^®^ posturography data in a representative subject**. Anterior–posterior deflections of the center of gravity (COG) for five trials are overlaid in each sensory organization test (SOT). **(A–F)** Indicate deflections of the COG in SOT1–6. **(G)** Shows a comparison of the equilibrium scores among the six SOT conditions. ***, **, *Significant difference between different SOT conditions in *post hoc* tests at *P* < 0.001, *P* < 0.01, and *P* < 0.05, respectively.

Figure 3 depicts anterior–posterior body sway in a representative subject under each SOT condition. Anterior–posterior deflections of the center of gravity in five trials are overlaid for each SOT condition (Figures 3A–F). The data indicated stronger deflections in SOT4–6 than in SOT1–3. Repeated-measures one-way ANOVA with Greenhouse–Geisser adjustment indicated that a significant main effect of the SOT was found [*F*(5,50) = 28.348, *P* < 0.001]. *Post hoc* tests with the Fisher protected least significant difference test indicated that anterior–posterior deflections were significantly stronger in SOT4–6 than in SOT1–3 and were stronger in SOT5 and 6 than in SOT4 (Figure 3G).

### Hemodynamic Responses to SOT

**Figure 4:**
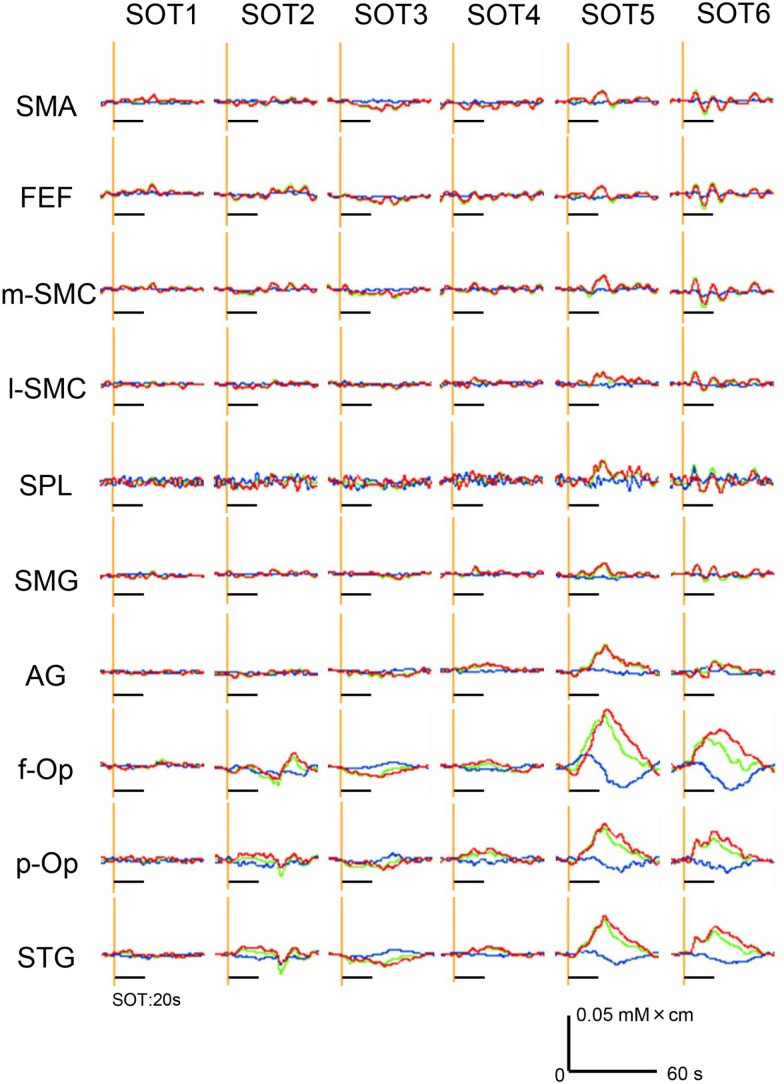
**Examples of the hemodynamic responses (changes in Oxy-Hb, Deoxy-Hb, and total Hb concentrations) in all 10 regions of interest (ROIs) under the six sensory organization test (SOT) conditions in a representative subject**. Red, green, and blue lines indicate changes in Oxy-Hb, total Hb, and Deoxy-Hb levels, respectively. Black horizontal bars indicate the 20-s time periods of the SOT.

Hemodynamic responses in a representative subject in 10 ROIs under the various SOT conditions are shown in Figure 4. Oxy-Hb and total Hb concentrations in the f-Op, p-Op, and STG around the Sylvian fissure were increased under SOT2, 3, 5, and 6, and especially under SOT5 and 6. These Oxy-Hb and total Hb responses were gradually decreased after the end of the task. Recognizable hemodynamic responses were not found in any of the ROIs under SOT1.

### Statistical Analyses of the NIRS Data

To analyze the statistical significance of the hemodynamic responses among the SOTs, the effect sizes in Oxy-Hb concentration were analyzed by two-way ANOVA (SOT × ROI). A significant main effect of ROI [*F*(9,90) = 16.128, *P* < 0.001, *ϵ* = 0.311] and a significant interaction of SOT × ROI [*F*(45,450) = 5.658, *P* < 0.001, *ϵ* = 0.116] were observed.

**Figure 5:**
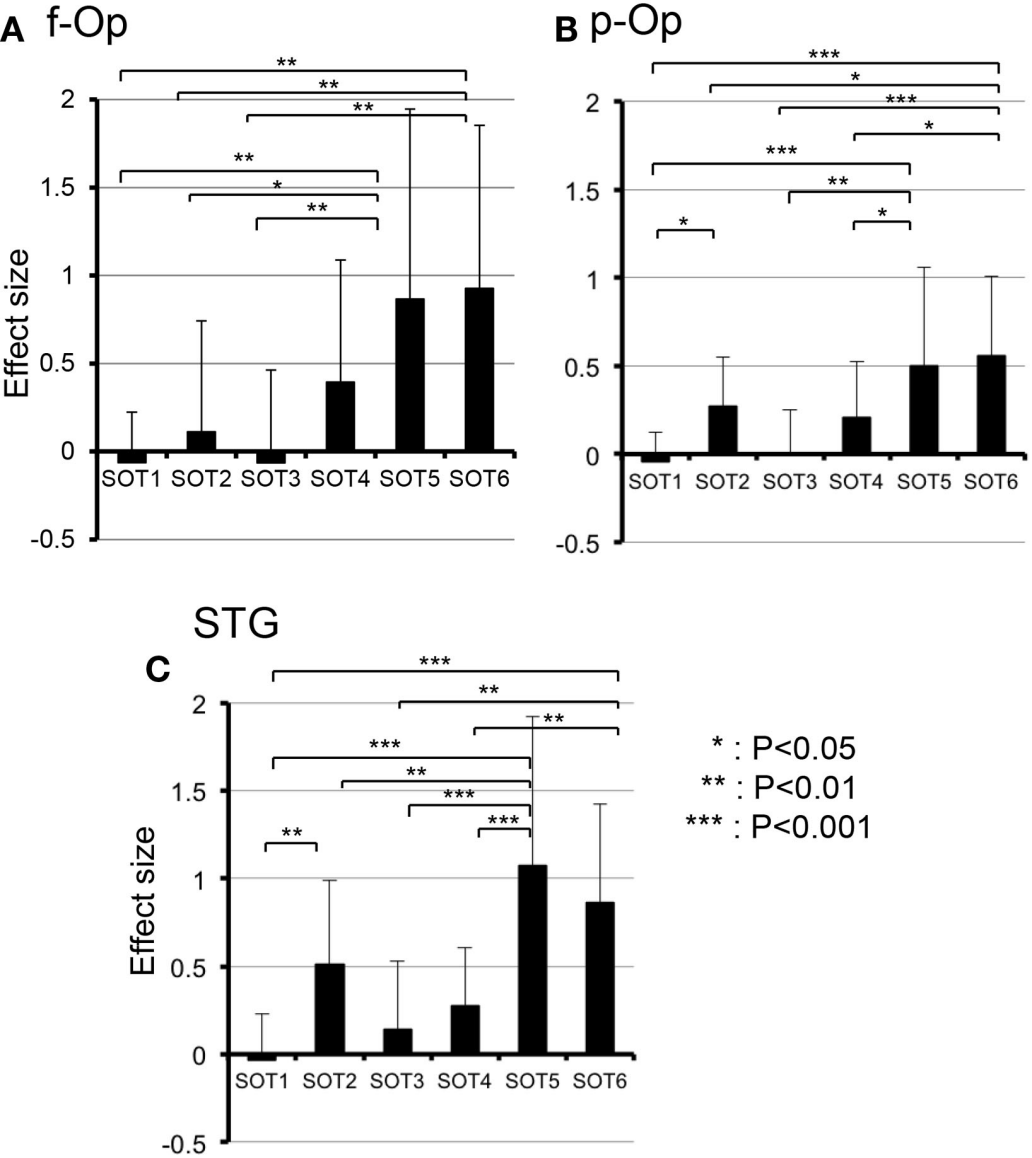
**Comparison of hemodynamic responses [effect sizes in Oxy-Hb concentration during each sensory organization test SOT)] among the six SOT conditions in the right frontal operculum/inferior frontal gyrus (f-Op) (A), right parietal operculum (p-Op) (B), and right superior temporal gyrus (STG) (C)**. These three ROIs indicated significant main effects of the SOT conditions in subsidiary statistical analyses after two-factorial ANOVA with repeated-measures (SOT condition × ROI). ***, **, *Significant difference between different SOT conditions in *post hoc* tests at *P* < 0.001, *P* < 0.01, and *P* < 0.05, respectively.

Subsidiary statistical analyses performed using one-way ANOVA in individual ROIs indicated that significant main effects of the SOT conditions were found in the p-Op [*F*(5,50) = 5.992, *P* < 0.001], f-Op [*F*(5,50) = 4.684, *P* < 0.01], and STG [*F*(5,50) = 9.603, *P* < 0.001]. *Post hoc* multiple comparisons using a Fisher protected least significant difference test indicated that in f-Op, the hemodynamic activities under SOT5 and 6 were significantly larger than those under SOT1-3 (Figure 5A). In p-Op, the hemodynamic activities under SOT5 and 6 were significantly larger than those under the SOT1, 3, and 4 (Figure 5B). Furthermore, that under SOT2 was significantly larger than that under SOT1 and smaller than that under SOT6 (Figure 5B). In the STG, the hemodynamic activities under SOT5 and 6 were significantly larger than those under SOT1, SOT3, and SOT4 (Figure 5C). Furthermore, the activity under SOT2 was significantly larger than that under SOT1 and smaller than that under SOT5 (Figure 5C).

**Figure 6:**
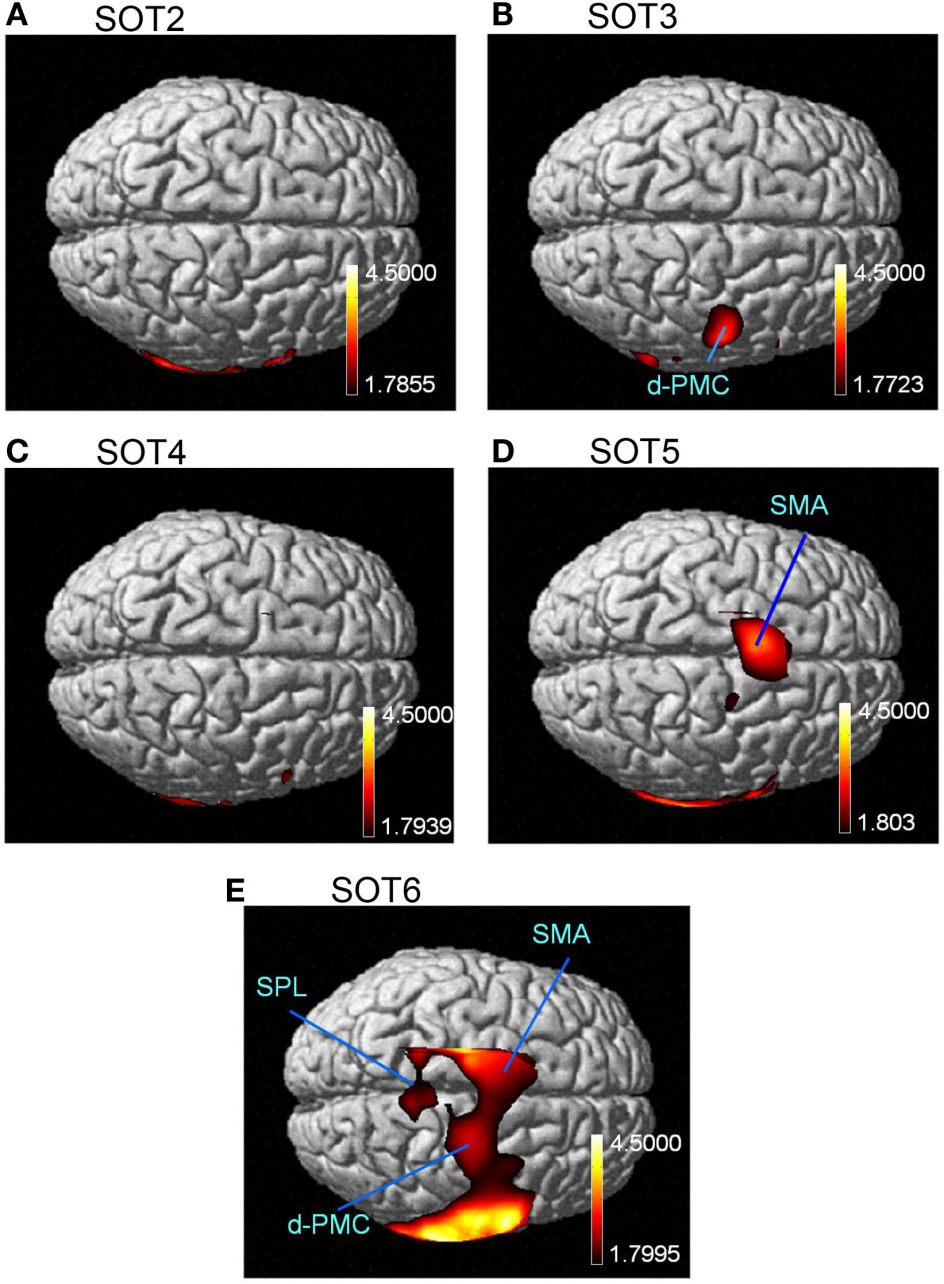
**The results of the group analyses using NIRS-SPM shown in the top views of the normalized brain surface**. The cortical regions are activated under sensory organization test (SOT) conditions 2–6 (A-E). The color scales represent the statistical significance of the T-values. d-PMC, dorsal premotor cortex in the right hemisphere; SPL, superior parietal lobule; SMA, supplementary motor area.

**Figure 7:**
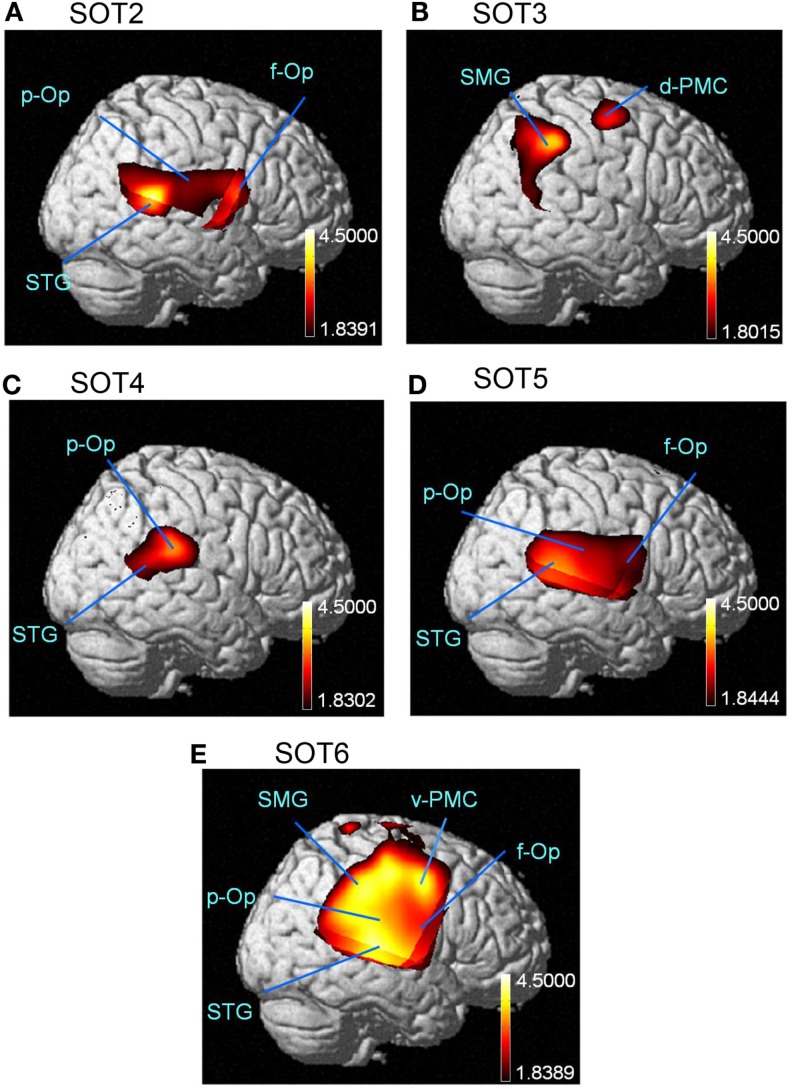
**The results of the group analyses using NIRS-SPM are shown in the right-side views of the normalized brain surface**. The cortical regions are activated under sensory organization test (SOT) conditions 2–6 (A-E). The color scales represent the statistical significance of the T-values. f-Op, right frontal operculum/inferior frontal gyrus; p-Op, right parietal operculum; SMG, right supramarginal gyrus; STG, right superior temporal gyrus; d-PMC, dorsal premotor cortex in the right hemisphere; v-PMC, ventral premotor cortex in the right hemisphere.

The results of the group statistical analyses based on the GLM with NIRS-SPM are shown in Figure 6 (top view) and Figure 7 (side view). The statistical results are also listed in Table 2. The topographical maps indicated significant activation in the right p-Op and STG under SOT2, 4, 5, and 6. The right f-Op was also activated under SOT2, 5, and 6. Under SOT3, the right SMG and the right dorsal premotor cortex (d-PMC) were activated. A significant activation in the SMA was also observed under SOT5 and 6. Furthermore, the right ventral premotor cortex (v-PMC) and d-PMC were also activated under SOT6.

**Table 2:** **Significantly activated cortical regions in the six SOT conditions in group analyses using NIRS-SPM**.

		Side	View	SOT1		SOT2		SOT3		SOT4		SOT5		SOT6	
Frontal lobe	f-OP	R	R			+	2.9231					+	2.1462	+	2.9506
	v-PMC	R	R											+	3.7418
	d-PMC	R	T					+	2.6782					+	3.3947
	SMA	R/L	T									+	3.1887	+	2.8244
Temporal lobe	STG	R	R			+	3.5145			+	3.1685	+	3.1913	+	4.0809
Parietal lobe	p-Op	R	R			+	2.4061			+	3.3087	+	2.7456	+	3.6151
	SMG	R	R					+	3.5711					+	3.9853
	SPL	R	T											+	2.8254

*“View” indicates direction of the statistical map in which the activated cortical region and *T*-value were analyzed. SOT, sensory organization test; NIRS-SPM, near-infrared spectroscopy-statistical parametric mapping; f-Op, frontal operculum/inferior frontal gyrus; v-PMC, ventral premotor cortex; d-PMC, dorsal premotor cortex; SMA, supplementary motor area; STG, right superior temporal gyrus; p-Op, parietal operculum; SMG, supramarginal gyrus; SPL, superior parietal lobule; R, right; L, left; T, top; +, statistically significant activation in a given condition. The number beside the + symbol indicates the *T*-value of group analyses using NIRS-SPM*.

### Functional Connectivities Among Activated Cortical Regions

**Figure 8:**
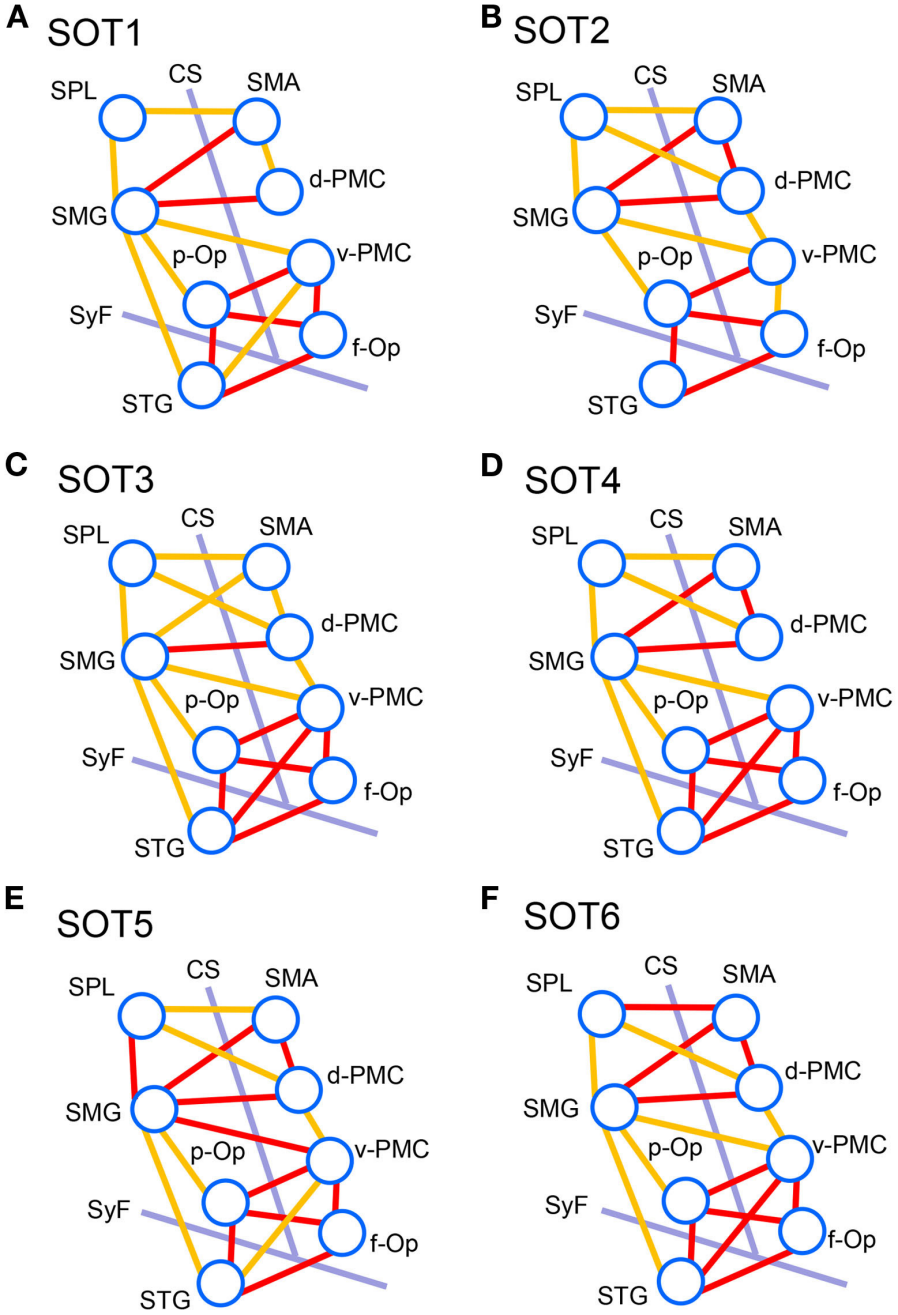
**Schematic presentation of functional connectivity maps among the activated cortical regions in NIRS-SPM under the six sensory organization test (SOT) conditions**. (A-F) indicate functional connectivity maps in SOT 1–6, respectively. The orange and red lines show correlations > 0.6 and 0.7 (averaged across all participants), respectively. f-Op, right frontal operculum/inferior frontal gyrus; p-Op, right parietal operculum; SMG, right supramarginal gyrus; STG, right superior temporal gyrus; d-PMC, dorsal premotor cortex in the right hemisphere; v-PMC, ventral premotor cortex; SPL, superior parietal lobule; SAC, somatosensory association cortex; SMA, supplementary motor area; CS, central sulcus in the right hemisphere; SyF, Sylvian fissure in the right hemisphere.

We analyzed functional connectivities between activated cortical regions using group statistical analysis with NIRS-SPM (i.e., f-Op, d-PMC, v-PMC, SMA, SPL, SMG, p-Op, and STG). The highest coefficients in all 28 pairs of regions were selected as functional connectivities in each subject. Figure 8 illustrates pairs of regions greater than a threshold (*r* > 0.6) in each SOT. There were high functional connectivities between cortical regions around the Sylvian fissure and the v-PMC. Furthermore, there were high connectivities between the parietal cortical regions (i.e., SPL and SMG) and frontal cortical regions (i.e., SMA, d-PMC, and v-PMC). The networks to maintain postural balance during the SOT showed almost the same patterns in all six SOT conditions (Figure 8).

**Figure 9:**
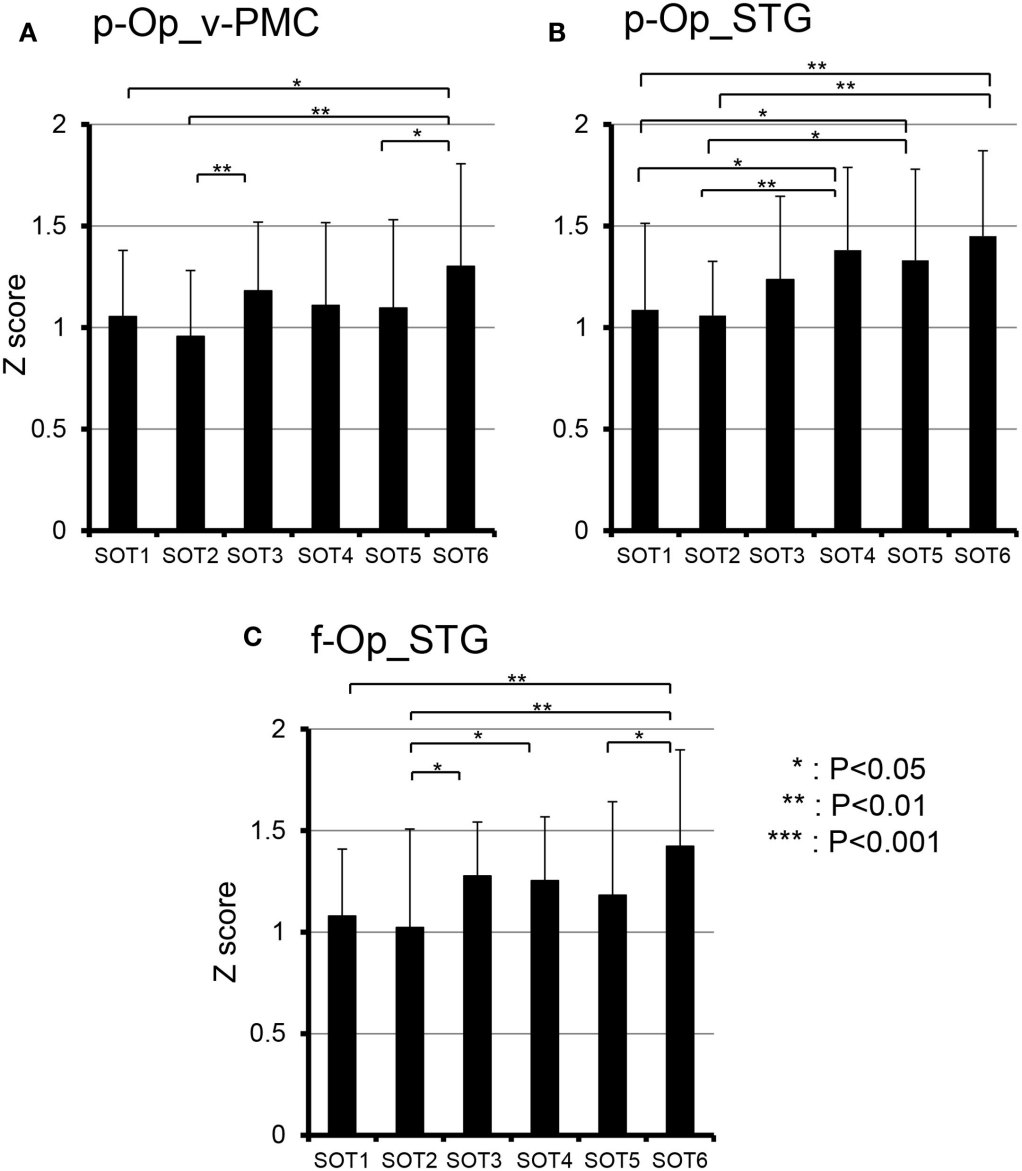
**Comparison of functional connectivities among the six sensory organization test (SOT) conditions in the p-Op and v-PMC (p-Op_v-PMC) (A), p-Op and STG (p-Op_STG) (B), and f-Op and STG (f-Op_STG) (C) pairs**. These three pairs indicated significant main effects of the SOT conditions in subsidiary statistical analyses after two-factorial ANOVA with repeated-measures (SOT × pair of regions). ***, **, *Significant difference between different SOT conditions in *post hoc* tests at *P* < 0.001, *P* < 0.01, and *P* < 0.05, respectively.

To analyze the statistical significance of the functional connectivities among the six SOT conditions, two-way ANOVA (SOT × region pair) was performed. There was a significant main effect of region pair [*F*(27,270) = 22.731, *P* < 0.001] and a significant interaction of SOT × region pair [*F*(135,1350) = 1.725, *P* < 0.001]. Subsidiary one-way ANOVA in individual region pairs indicated that significant main effects of the SOT conditions were found in the pairs between p-Op and v-PMC (p-Op_v-PMC) [*F*(5,50) = 2.852, *P* < 0.05], between p-Op and STG (p-Op_STG) [*F*(5,50) = 4.105, *P* < 0.01], and between f-Op and STG (f-Op_STG) [*F*(5,50) = 3.233, *P* < 0.05]. *Post hoc* multiple comparisons indicated that in p-Op_v-PMC, the *z* score was significantly larger in SOT6 than in SOT1, 2, and 5 (Figure 9A). In p-Op_STG, the *z* scores were significantly larger in SOT5 and 6 than in SOT1 and 2 (Figure 9B). In f-Op_STG, the *z* score was significantly larger in SOT6 than in SOT1, 2, and 5 (Figure 9C). Furthermore, the *z* score was significantly larger in SOT3 and 4 than in SOT2 (Figure 9C).

## Discussion

The present study indicated the occurrence of activations in the right temporal-parietal areas (STG and SMG) when both vision and proprioceptive information were degraded, consistent with a previous study (Karim et al., 2013). Furthermore, the present results indicated activation of the broader cortical networks for vestibular information processing, spatial cognition, and motor control and learning, which might be essential for postural balance control, during CDP (see below).

### Cortical Regions Around the Sylvian Fissure

In the present study, changes in Oxy-Hb concentration were significantly increased in the right f-Op, right p-Op, and right STG under SOT5 and 6. Furthermore, group statistical analyses by NIRS-SPM indicated significant activation in the right p-Op and STG under SOT2, 4, 5, and 6 and in the right f-Op under SOT2, 5, and 6. These results strongly suggest that cortical activities around the Sylvian fissure were required to maintain postural stability in SOT2, 4, 5, and 6. The posturography data indicated that anterior–posterior deflections of the body were significantly increased in SOT5 and 6 (Figure 3). These changes are consistent with the increase in Oxy-Hb concentration in SOT5 and 6. These results suggest that severe postural instability in sensory conflict activates the right p-Op, f-Op, and STG.

Previous animal (Fredrickson et al., 1966; Odkvist et al., 1974; Faugier-Grimaud and Ventre, 1989; Guldin and Grüsser, 1998) and human (Lobel et al., 1998; Bense et al., 2001; Bottini et al., 2001; Fasold et al., 2002; Dieterich et al., 2003; Eickhoff et al., 2006; Dieterich and Brandt, 2008) studies indicated that these three cortical regions (p-Op, f-Op, and STG) are parts of the multisensory vestibular cortical areas. In particular, p-Op is a homolog of the parieto-insular vestibular cortex (PIVC) that has been postulated to be a core region within the vestibular cortical system with its strong interconnections with other vestibular cortical areas (Brandt and Dieterich, 1999; Eickhoff et al., 2006). These results strongly suggest that cortical activities around the Sylvian fissure reflect activations in the vestibular cortical areas in SOT2, 4, 5, and 6.

Extensive studies reported that the cortical areas related to high-level cognitive processing are involved in shaping the postural responses evoked by external postural perturbations (Miyai et al., 1997; Woollacott and Shumway-Cook, 2002; Jacobs and Horak, 2007; Maki and McIlroy, 2007). Using EquiTest^®^, Miyai et al. (1997) investigated the location of supratentorial strokes associated with impaired standing balance (ISB) that were not ascribed to hemiparesis, proprioceptive deficits, or visual-vestibular abnormality. The patients who could stand under SOT1 but not under SOT5 were designated as the ISB group. A second group of the patients who could maintain their standing balance during both SOT1 and 5 were selected as controls (control group). Most patients in the ISB group had lesions in the insula or adjacent structures including the STG, f-Op, subinsular white matter, and putamen, whereas the control group had no lesions in these areas. These areas are perfectly congruent with the activated cortices in the present study.

It is now generally accepted that visual, vestibular, and somatosensory inputs are dynamically reweighted to maintain upright stance as environmental or nervous system conditions change, a phenomenon referred to as sensory reweighting (Assländer and Peterka, 2014; Logan et al., 2014). Human functional imaging studies suggest that there is a reciprocally inhibitory interaction between the vestibular and visual areas during self-motion perception and that this mechanism allows a shift of the dominant sensorial weight from one sensory modality (vestibular or visual) to the other during self-motion perception (Brandt et al., 1998; Deutschländer et al., 2002; Bense et al., 2005). In balance control during perturbation, sensory information is delivered to the CNS as feedback, and the sensory inputs must be integrated. A few motor command signals from the CNS are generated to coordinate multiple spatial patterns of muscle activations, which is called “muscle synergy” (Lockhart and Ting, 2007; Ting, 2007). A recent study suggests that a group of weighted sensory inputs, called “sensory synergy,” are integrated and recruited to the CNS to simplify the construction of muscle synergy (Alnajjar et al., 2015). Thus, the process of sensory reweighting might have an essential role in maintaining postural balance in sensory conflict.

Under SOT2, 4, 5, and 6, vestibular inputs were normal, whereas either somatosensory or visual inputs or both were unusual for the participants. The participants presumably suppressed inaccurate sensory information during sensory conflicts by online recognition of spatial orientation and perception of self-motion from correct sensory information. They might set a high value on vestibular inputs as the correct sensorial inputs under SOT2, 4, 5, and 6, and therefore vestibular cortices would be activated. However, neural mechanisms for dynamic selection of appropriate sensory information during sensory conflict remain unclear. Further studies are required to clarify neural mechanisms of visual–vestibular–somatosensory interaction on posture during sensory conflict.

### Frontal and Posterior Parietal Cortices

The group statistical analyses with NIRS-SPM also indicated significant activation in the SMA under SOT5 and 6. Previous studies suggested that the SMA is involved in establishing motor programs (Picard and Strick, 1996), preparation for foot movement (Sahyoun et al., 2004), human locomotion (Miyai et al., 2001), and human balance control (Mihara et al., 2008). Hardwick et al. (2013) performed a meta-analysis to identify consistent activations across 70 motor learning experiments using activation likelihood estimation. Their results indicated that the area homologous to the SMA in the present study was activated in all motor learning tasks.

Under SOT5 and 6, vestibular inputs were normal, whereas somatosensory and visual inputs were unusual or absent. The combination of these three inconsistent sensory inputs under SOT5 and 6 might induce unpredictable perturbations in postural equilibrium. In such situations, the subjects might not move their body automatically and might not use the previously acquired motor programs to maintain postural balance. Therefore, they might have to execute volitional action and establish new motor programs to maintain postural balance. The SMA might be activated under SOT5 and 6 for these purposes.

In the present study, the PPC, including the SPL, SMG, and ventral/dorsal PMC, was activated under SOT3 and 6. In primates and humans, the PPC consists of the SPL and the inferior parietal lobule (IPL). The human SPL consists of Brodmann areas 5 and 7, whereas the IPL consists of the SMG and AG. Previous studies suggest that the PPC is involved in spatial cognition (Sack, 2009) and particular visuomotor actions such as reaching, grasping, and eye movements (Culham and Valyear, 2006). In addition, the human PPC is recruited during the processing and perception of action-related information (Culham and Valyear, 2006).

The PPC is suggested to have strong connections with the PMC (Rizzolatti and Matelli, 2003; Averbeck et al., 2009; Sack, 2009). The dorsal and ventral PMC are involved in selecting and planning motor behaviors and in preparing and executing movements (Hoshi and Tanji, 2007). The PPC receives visual and somatosensory inputs and sends afferents to the PMC. The SPL projects to the primary motor cortex and dorsal PMC, whereas the IPL projects to the ventral PMC and prefrontal cortex (Rizzolatti and Matelli, 2003). Sack et al. (2008) indicated a dynamic interaction between the PMC and PPC during spatial imagery. Previous studies implicate the PPC and PMC in spatial coding, updating of spatial information (Zaehle et al., 2007), and the computation of spatial reference frames (Fink et al., 2003).

Humans represent behaviorally relevant information in different spatial reference frames (e.g., eye-centered for saccades, body-centered for limb movements, object-centered for certain cognitive manipulations, and world-centered for navigation) to interact effectively with the environment (Szczepanski and Saalmann, 2013). The connection between the PPC and PMC is vital for performing transformations between these different coordinate systems (Szczepanski and Saalmann, 2013). Under SOT3 and 6, both vision and proprioception were compromised, leaving the vestibular system as the only reliable source of information. Sensory conflict between the vestibular and visual information might cause sensorial reweighting to vestibular inputs, resulting in flexible transformation of the spatial reference frame from allocentric (e.g., world-centered) to egocentric (body-centered). Thus, the PPC and PMC were activated under SOT3 and 6.

Recently, the association between multisensory bodily stimuli and conscious aspects of the self has been investigated, and different components of bodily self-consciousness have been identified during multisensory conflicts (Ionta et al., 2014). In particular, visuo-tactile conflict has been used to manipulate the sense of body ownership (Blanke, 2012). It has been accepted that there are three important aspects of bodily self-consciousness: self-identification with body (the experience of owning a body), self-location (the experience of where I am in space), and first-person perspective (the experience from where I perceive the world) (Blanke, 2012). Self-location and first-person perspective are induced by visuo-tactile conflict and depend on visuo-tactile signals and their integration with vestibular signals (Blanke, 2012). Functional imaging studies have indicated that illusory self-identification with the virtual body is associated with activity in the bilateral v-PMC, left intraparietal sulcus (i.e., a part of the PPC), and the left putamen and that illusory self-location and first-person perspective are associated with the bilateral posterior STG, right temporoparietal junctions (TPJ), primary somatosensory cortex and medial PMC, and the adjacent prefrontal cortex (Blanke, 2012). Most activated cortical regions in the Blanke (2012) study overlap with those in our studies (i.e., TPJ close to the p-Op, STG, PMC, and PPC). Thus, the brain activation pattern in the situation of bodily self-consciousness in the Blanke (2012) study is very similar to that in the SOT task. In the SOT task, participants had to consciously perceive their body images in space during the maintenance of postural balance in sensory conflict. The activations in the p-Op, STG, PMC, and PPC in the present study might reflect cognitive processes of bodily self-consciousness.

### Functional Connectivities Between Frontal Cortex, PPC, and Cortical Regions Around the Sylvian Fissure

We also found strong functional connectivities between the peri-Sylvian regions (i.e., the p-Op, f-Op, and STG), suggesting that this network around the Sylvian fissure processes vestibular information during the maintenance of postural balance. A meta-analysis of fMRI and PET studies using vestibular stimulus and functional connectivity analysis indicated that the right hemispheric parietal area OP2 (p-Op 2), which is homologous to the PIVC in animal studies, is implicated as a core region for vestibular processing and that the OP2 has a direct connection with temporoparietal regions, the PMC, and the midcingulate gyrus (zu Eulenburg et al., 2012). The authors suggest that the functional connectivity networks between the OP2, temporoparietal regions, and midcingulate gyrus are analogous to the network previously identified in animal studies (Guldin and Grüsser, 1998), termed the *inner vestibular circle*. Another study with resting-state functional connectivity analysis in patients with chronic bilateral vestibular failure revealed stronger connectivity from the right posterior insula (i.e., close to the p-Op in the present study) to the anterior insula (i.e., close to the f-Op in the present study), ACC, precuneus, and middle frontal gyrus (Göttlich et al., 2014).

A recent study investigated network activities associated with self-location and first-person perspective and revealed that bilateral TPJ, close to the p-Op, are bilaterally connected to the SMA, v-PMC, insula, intraparietal sulcus, and occipitotemporal cortex (Ionta et al., 2014). The v-PMC is associated with illusionary body ownership due to multisensory conflicts, especially between visual and somatosensory inputs (Petkova et al., 2011). The authors suggest that processing of self-related multisensory bodily information recruits a bilateral network centered at the TPJ that includes the premotor, intraparietal, and occipitotemporal cortices (Petkova et al., 2011). Another study on conflict between vision and proprioception using fMRI indicated that the bilateral PMC and right TPJ were activated during monitoring of incongruent compared with congruent movements, suggesting an interaction between vision and proprioception in orienting to special locations (Balslev et al., 2005).

The present study on functional connectivity indicated higher connectivity (*r* > 0.7) among the cortices around the Sylvian fissure (i.e., p-Op, f-Op, and STG) and v-PMC in SOT1-6 (Figure 8). The present SOT task also induced multisensory conflicts among vestibular, visual, and somatosensory inputs as in the previous studies on bodily self-consciousness (see above). The connectivity network between the peri-Sylvian regions and v-PMC in the present study might be involved in the monitoring and detection of multiple sensory conflicts and sensory reweighting. In addition, these cortical network activities may be variable depending on the cognitive demands in sensory conflict because connectivities among the p-Op, f-Op, and v-PMC were significantly different under different SOT conditions (Figure 9). In particular, functional connectivity between the p-Op and v-PMC may be crucial to the detection of vestibulo-visual conflict in the present study because the *z* scores under SOT2 and 5 with the eyes closed (i.e., visual inputs were absent) were significantly smaller than those under SOT3 and 6 (i.e., the vestibular and visual inputs were incongruent) (Figure 9A).

In the connectivity analysis, we found higher connectivity between the PPC (SMG and SPL) and the frontal cortical regions (SMA, d-PMC, and v-PMC) (Figure 8). Furthermore, the SMG had significant connectivity with peri-Sylvian regions (p-Op and STG) (Figure 9). The present results suggest that the SMG is a hub connecting these regions. The statistical analyses did not indicate significant differences in the magnitudes of these connectivities among the SOT conditions, suggesting that the network between the PPC and frontal cortical regions is constitutively active during postural balance regardless of sensory conflict.

The PPC is known to be a site of sensorimotor integration. In the macaque, the ventral intraparietal area (VIP), located in the fundus of the intraparietal sulcus, receives vestibular inputs from the PIVC (i.e., the core region of the inner vestibular circle), somatosensory, and vestibular inputs from the neck and vestibular subregions of areas 3a and 2 (Guldin et al., 1992; Lewis and Van Essen, 2000), visual inputs from the medial temporal area and the medial-superior temporal complex (Lewis and Van Essen, 2000), and somatosensory inputs from the primary somatosensory cortex (Lewis and Van Essen, 2000). Thus, multimodal sensory inputs converge on the PPC and are used for the analysis and encoding of self-motion (Bremmer et al., 2002). A human fMRI study indicated the existence of the human equivalent of the macaque VIP area in the depth of the intraparietal sulcus (Bremmer et al., 2001). As mentioned in the preceding section, the PPC has a strong connection with the PMC (Rizzolatti and Matelli, 2003; Averbeck et al., 2009; Sack, 2009). Multiple pathways exist in the human brain, each of which connects areas in the PPC to either motor, premotor, or SMAs in the frontal cortex to facilitate special guided action (Szczepanski and Saalmann, 2013). The strong connectivities between the PPC and frontal regions may indicate a network for integrative roles in polymodal motor processing in sensory conflict. Convergence of connectivity to the SMG may indicate that the function of the SMG is to integrate the multimodal reweighted sensory information and recruit it to the motor systems.

From the above results, we suggest that two cortical networks, one among the peri-Sylvian cortices and v-PMC (peri-Sylvian network) and one between the PPC and frontal cortex (parieto-frontal network), are involved in postural balance in a situation of sensory conflict. These two networks converge on the SMG. The peri-Sylvian network may function to detect sensory conflict and sensory reweighting and send the reweighted sensory information to the SMG. The parieto-frontal network may function for integration based on reweighted sensory information, spatial reorientation, and the selection and planning of motor behaviors.

## Conclusion

Hemodynamic activity was significantly increased in the f-Op, right p-Op, and right STG under SOT2, 4, 5, and 6. These activated cortical regions have been reported as the vestibular cortices in previous studies. These findings suggest that the dominant sensorial weight was shifted to the correct sensory inputs (i.e., vestibular inputs) during sensory conflict under SOT2, 4, 5, and 6 and that the cortical areas related to the recognition of spatial orientation and the perception of self-motion were activated to maintain postural balance during unpredictable perturbations.

Furthermore, the hemodynamic activity in the SMA was significantly increased under SOT5 and 6, suggesting that the SMA might be activated for volitional action control and establishment of new motor programs to maintain postural balance. In addition, the hemodynamic activity in the PPC and PMC was significantly increased under SOT3 and 6. These results suggest the involvement of these areas in the updating and computation of spatial reference frames during instances of sensory conflict between vestibular and visual information.

Alnajjar et al. (2015) proposed a conceptual model of a neural sensorimotor synergy system in balance control during perturbation, which includes sensory synergy, neural command processing, and muscle synergy. Based on the present results, it is possible that the peri-Sylvian network is responsible for sensory synergy, and the parieto-frontal network is responsible for neural command processing and muscle synergy. Further studies are required to clarify the neural mechanisms responsible for the dynamic shift of brain activation during sensory conflict.

## Conflict of Interest Statement

Akihiro Ishikawa is an employee of Shimadzu Co., Ltd., who made the NIRS apparatus used in this study. Hiromasa Takakura, Hisao Nishijo and Hideo Shojaku have no conflict of interest to declare.
